# Sex Differences in Acute Coronary Syndromes: A Global Perspective

**DOI:** 10.3390/jcdd9080239

**Published:** 2022-07-27

**Authors:** F Aaysha Cader, Shrilla Banerjee, Martha Gulati

**Affiliations:** 1 Department of Cardiology, Ibrahim Cardiac Hospital & Research Institute, Dhaka 1000, Bangladesh; aaysha.cader@gmail.com; 2 Department of Cardiology, Surrey and Sussex Healthcare NHS Trust, Redhill RH1 5RH, UK; shrilla.banerjee@nhs.net; 3 Barbra Streisand Women’s Heart Center, Cedars-Sinai Smidt Heart Institute, 127 S. San Vicente Blvd, Suite A3206, Los Angeles, CA 90048, USA

**Keywords:** acute coronary syndrome, sex differences, cardiovascular disease, women, global

## Abstract

Despite increasing evidence and improvements in the care of acute coronary syndromes (ACS), sex disparities in presentation, comorbidities, access to care and invasive therapies remain, even in the most developed countries. Much of the currently available data are derived from more developed regions of the world, particularly Europe and the Americas. In contrast, in more resource-constrained settings, especially in Sub-Saharan Africa and some parts of Asia, more data are needed to identify the prevalence of sex disparities in ACS, as well as factors responsible for these disparities, particularly cultural, socioeconomic, educational and psychosocial. This review summarizes the available evidence of sex differences in ACS, including risk factors, pathophysiology and biases in care from a global perspective, with a focus on each of the six different World Health Organization (WHO) regions of the world. Regional trends and disparities, gaps in evidence and solutions to mitigate these disparities are also discussed.

## 1. Introduction: Global Burden of ACS and Disparities

Acute coronary syndromes (ACS) encompass a spectrum of clinical presentations, predominantly differentiated on the basis of the presenting electrocardiogram as either ST-segment elevation myocardial infarction (STEMI) or non-ST-segment elevation ACS (NSTEACS). The presentation is wide-ranging and includes cardiac arrest, electrical or hemodynamic instability with cardiogenic shock due to ongoing ischemia or mechanical complications such as severe mitral regurgitation to patients who are already pain-free again at the time of presentation [1]. Despite the increasing emphasis on sex and gender disparities in the presentation, management and outcomes of acute coronary syndromes (ACS), much of these data come from North America, Australia and Europe, where such research has predominantly been undertaken [2,3,4,5,6,7,8]. Indeed, there are few reports of sex disparities in ACS outcomes from certain regions of the world, particularly Africa [9], where the focus on non-communicable diseases (NCDs) has been less, given that resources have been primarily allocated towards communicable diseases [10]. Nonetheless, cardiovascular disease (CVD) remains the leading cause of mortality in women globally [11].

Amongst global ACS registries, smaller proportions of women are enrolled compared with men [12,13,14,15,16,17,18,19,20], and there are even less sex-disaggregated data based on the type of ACS, i.e., STEMI, non-ST segment elevation myocardial infarction (NSTEMI) or unstable angina (UA). Culturally, economically and in terms of healthcare delivery, there are vast differences across the world. In high-income countries, advanced research on ACS has been undertaken, including sex disparities in plaque morphology by intravascular ultrasound (IVUS) [21] and the use of advanced revascularization therapies [14]. In contrast, in resource-constrained settings, contemporary data are still limited to issues at a more grassroots level, such as access to care, delays in presentation, bias in guideline-directed medical therapy (GDMT) and varying socioeconomic and geo-cultural factors that contribute to these differences. Even where data on sex differences are present, the extent and causes of these differences in some parts of the world are poorly understood.

This review aims to summarize the existing and recent data on sex differences in ACS from a global perspective, with emphasis on specific regional trends, gaps in evidence and research recommendations where relevant.

## 2. Sex Differences in Presentation of ACS

Sex differences are the biological and physiological differences in the cardiovascular system that are a result of different gene expressions due to sex chromosomes. Some well-documented sex differences in ACS include that women are older and have more comorbidities when compared to men [6,22,23].

Timely recognition of ACS is essential to the timely initiation of therapies and ultimately affects the outcomes of ACS. Delayed recognition of ACS in both patients and providers has contributed to delays in treatment initiation and outcomes [24,25]. This has been in part due to the continued message that women with ACS present with “atypical” symptoms based on retrospective data [26,27,28]. Nonetheless, there is extensive overlap in symptoms based on sex, and in fact, more contemporary literature has demonstrated that symptoms in women are often more similar to men, [29,30] with a number of studies demonstrating that approximately 90% of women and men with a myocardial infarction (MI) reporting experiencing chest pain/pressure symptoms as their presenting symptom [31,32]. There were some noted sex differences in symptoms of ACS, being that women often report three or more accompanying symptoms along with the chest discomfort that they experience [31,32]. As a result of these recent findings, the 2021 Chest Pain Guidelines from the American Heart Association and the American College of Cardiology have a Class 1 recommendation to no longer use the word “atypical” to describe chest pain, in addition to recognizing that accompanying symptoms are more common in women with ACS [33]. Women with chest pain continue to be underdiagnosed for ACS and have less timely and appropriate care. Additionally, the use of the word “atypical” to describe chest pain often fosters less intensive care. The goal of these recommendations is to reduce the sex gaps in the care of ACS.

## 3. Pathophysiology of Acute Coronary Syndromes in Women

The pathophysiology of ACS presentations in women is complex and multifaceted. Some presentations are associated with traditional risk factors (RF), some with non-traditional RFs, and some as the result of sex-specific risk enhancers [34].

### 3.1. Traditional Risk Factors

Women presenting with ACS are older and more likely to be diagnosed with diabetes, hypertension and congestive cardiac failure than men [4,5,6,12,17,35,36,37].

Traditional risk factors are often seen in women presenting with ACS, but the impact of individual components of risk appears to be more pronounced with respect to smoking, diabetes and obesity [38]. Indeed, the diagnosis of diabetes in a female ACS patient increases the risk of MI four-fold, whereas, in a man, it only increases the MI risk 2.5 times [39]. The relative risk of an MI in a female smoker is 3.3 versus 1.9 in a male smoker [40]. Similar impacts are seen with hypertension and obesity. A maternal family history of MI at an age less than 65 years is associated with a four-fold increase in ACS in the female offspring when compared to other women of the same age or older [41].

### 3.2. Non-Traditional Risk Factors

The impact of non-traditional risk factors is poorly understood, but it is known that women presenting with ACS are likely to have lower socioeconomic state (SES) and quality of life (QOL) scores and higher psychosocial burdens [42]. Low SES impacts women more than men with an ACS presentation [43]. Stress is known to have an association with premature cell “weathering” that is hypothesized to be responsible for the poor outcomes for ACS in patients suffering from race-based discrimination [44].

### 3.3. Female Specific Risk Enhancers

The 2018 American College of Cardiology (ACC)/American Heart Association (AHA) guidelines on the Management of Blood Cholesterol includes the use of risk enhancers in risk assessment, some of which are sex-specific [45]. Uniquely, only biological women may experience pregnancy, and pregnancy is a cardiovascular stress test, identifying women at an increased risk of the development of CVD [34,46]. Women who experience adverse pregnancy outcomes (APOs), including hypertension during pregnancy (including preeclampsia and eclampsia), gestational diabetes, pre-term birth, and small for gestational age births are all associated with increased CVD [47,48]. Indeed, gestational diabetes is associated with a 2.5 times increased risk of CAD [49,50] and preterm delivery 1.5 times [48]. The inclusion of gestational complications in risk scores is seen in the increased predictive accuracy [51]. Premature menopause, especially natural premature menopause, is known to be independently associated with clonal hematopoiesis of indeterminate potential (CHIP) and serves as a risk factor for CAD [52]. Polycystic ovary syndrome, whose pathogenesis involves insulin resistance leading to cardiometabolic abnormalities including dyslipidemia, hypertension, glucose intolerance, diabetes and metabolic syndrome, also puts women at an increased risk for CVD, particularly CAD [53,54].

## 4. Female Coronary Anatomy and Physiology

Women are known to have smaller epicardial coronary arteries, even after correction for age, body habitus and LV mass [55]. However, in spite of smaller vessels, the presence of higher baseline myocardial flow results in an equivalent coronary flow reserve (CFR) for men [56]. As a consequence, the coronary vessels of female patients are then susceptible to higher endothelial shear stress, which may result in a difference in susceptibility to coronary artery disease (CAD) [57].

## 5. Myocardial Infarction with Non-Obstructive Coronary Artery (MINOCA) Presentations

An important and understudied group are patients with MINOCA (5–15% of all patients with a clinical diagnosis of MI). MINOCA is an umbrella term that incorporates patients with acute myocardial infarction (AMI) with non-obstructive coronary arteries on angiography and no specific alternate diagnosis for the clinical presentation [1] MINOCA encompasses different causations, including those with less than 50% stenoses on angiography, coronary microvascular dysfunction (CMD) and vasomotor dysfunction, spontaneous coronary artery dissection (SCAD) and coronary thromboembolism [1,58,59].

The diagnosis of MINOCA can be enhanced by performing a comprehensive invasive coronary assessment, including intravascular imaging and coronary physiology assessment, although cardiovascular magnetic resonance (CMR) and computerized tomography (CT) and thrombophilia assessments are also complementary [1,60].

Women with MINOCA ACS presentations (38% of all MINOCA patients) undergoing coronary angiography and intravascular imaging have more diffuse, non-obstructive disease and more plaque erosion than plaque rupture, which is more commonly seen in men [60,61,62,63]. 

Coronary microvascular dysfunction accounts for 32% of patients presenting with MINOCA. CMD can occur secondary to changes within the microcirculation or vasospastic behavior of the circulation. This may result in an inability to increase flow when demand increases, an impaired CFR, and is associated with an adverse prognosis [64].

SCAD is an uncommon cause of ACS (16% of all causes) [60], resulting from either a rupture of the vasa vasorum or an intimal tear, which then results in an intramural hematoma that compromises coronary flow. Of patients presenting with STEMI as a result of SCAD, 90% are women. SCAD is an important differential to consider, as best management is often non-interventional. SCAD usually occurs in younger women (<50 years of age) and is responsible for up to 34% of ACS in women [65,66]. The risk of recurrence is 27% at 5 years [67]. 

Coronary thromboembolism (11% of all MINOCA causations) may result in angiographically normal coronary vessels following a transient occlusion resulting in STEMI. Emboli may originate from arterial or venous circulations. Hereditary disorders, including factor V Leiden thrombophilia and Protein S and C deficiencies, occur in up to 11–14% of these presentations [60,68]. Paradoxical embolism may be seen in patients with patent foramen ovale (PFO) or other septal defects [69,70]. Emboli may also occur in the setting of atrial fibrillation, myxomas and cardiac tumors.

An alternative presentation of MI without obstructive coronary artery disease that must be considered is Stress Cardiomyopathy (also known as Takotsubo cardiomyopathy or Broken Heart Syndrome), which is more frequently seen in women and must be considered when a working diagnosis of MINOCA is entertained [71]. These account for 11% of all myocardial infarctions without evidence of obstruction, but it remains controversial if this is a form of MINOCA [72,73] rather than a form of cardiomyopathy. This classically presents with a flask-shaped left ventricular chamber and transient LV dysfunction, usually after acute stress, whether emotional or physical event. The actual pathophysiological mechanism is uncertain and may include catecholamine-induced myocardial stunning and extreme microcirculatory dysfunction or even multivessel epicardial spasm [74]. Reversible CMD is often seen in association with Takotsubo presentations [75] regardless of etiology.

## 6. Bias in the Care of Women with STEMI

There remain significant disparities and bias in the cardiac care of women, particularly exemplified in the care of women presenting with AMI [76]. In particular, women who experience STEMI continue to have poorer care, which contributes to worse outcomes when compared to men. Delays in care, lack of GDMT, and inequalities in timely reperfusion therapies or any revascularization continue to be demonstrated globally [2,13,77,78,79,80,81,82,83,84,85,86,87]. These disparities in care are even more pronounced in women < 55 years [83,84], who have the highest mortality after AMI [84].

Despite the awareness of these sex treatment gaps after an AMI over decades, there is very little evidence showing improvement over time. In fact, there is some evidence that these disparities have continued to widen, particularly in revascularization and initiation of optimal lipid-lowering therapies [84]. Mortality rates after AMI are worse in women, particularly after STEMI, and even the implementation of protocol-driven initiatives to reduce sex gaps in STEMI care have not been able to completely improve the care of women or cardiovascular outcomes [85].

## 7. Sex Disparities in ACS: Global Trends 

**Figure 1:**
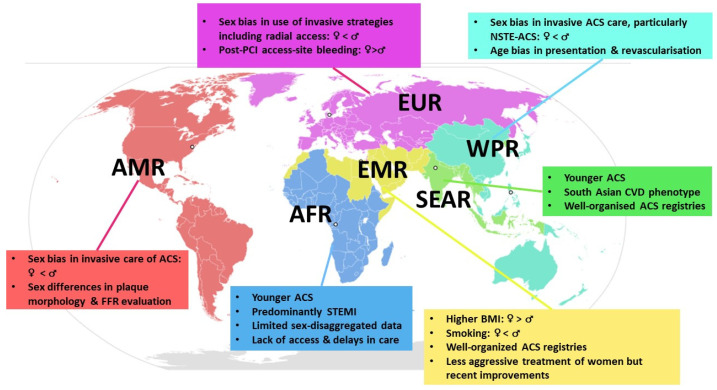
Sex disparities in acute coronary syndromes globally: Significant characteristics in each WHO region. *World Health Organization (WHO) regions; African Region (AFR); South-East Asian Region (SEAR); European Region (EUR); Eastern Mediterranean Region (EMR); Western Pacific Region (WPR). Acute coronary syndrome (ACS); Body Mass Index (BMI); Cardiovascular disease (CVD); Fractional Flow Reserve (FFR): Non-ST segment elevation acute coronary syndrome (NSTE-ACS); Percutaneous Coronary Intervention (PCI); ST-Segment elevation myocardial infarction (STEMI)*.

For purposes of reporting, analysis and administration, the World Health Organization (WHO) divides the world into six WHO regions, namely the African Region (AFR), Region of the Americas (AMR), South-East Asian Region (SEAR), European Region (EUR), Eastern Mediterranean Region (EMR) and Western Pacific Region (WPR) [88] Appendix A. A brief review of data from each region underscores not only sex disparities in ACS that exist globally but also regional disparities in ACS care and research (Figure 1) that are not adequately managed even in high-income countries. 

## 8. African Region (AFR)

Traditionally, there has been a focus on infectious diseases research in most of Sub-Saharan Africa (SSA), with a prior perception of low NCDs and, indeed, CAD mortality rates [89]. Although the increasing burden of ACS, and particularly STEMI, is being recognized in SSA, only small-scale data are available for most countries and much less on sex differences. 

A systematic review of ACS in ten countries in SSA reported a prevalence of 0.21% to 22.3% among patients admitted for CVD [9]. Overall, African patients are much younger than those in high-income countries [10], with an age difference that frequently reaches 10 years [90]. A contemporary national Registry for Acute Coronary Events in Nigeria (RACE Nigeria) reports a mean age of 59.2 ± 12.4 years [89]. Male predominance in registries was common. Similar to global trends, men presented about a decade earlier than women in frequency of ACS [89]; a high prevalence of CV risk factors was noted among women, particularly diabetes, hypertension and obesity [91,92], with a lower prevalence of smoking [92,93].

STEMI, comprising over half of all ACS presentations, was the predominant presentation in both sexes in many analyses [91,92], possibly reflecting the limited availability of diagnostic tools such as troponin, leading to a lower rate of documented MI cases, notably NSTEMI [9]. In RACE Nigeria, women presented with more NSTEMI [89].

Delays in the presentation were common for both sexes, with less than 10.9–11.2% presenting within the 12-h reperfusion window [89,92], reflecting the common use of non-medical transport to reach hospitals, where organized structures and ambulances may be lacking [9]. Women have been demonstrated to have longer door-to-admission times at the emergency department (ED) in Senegal, and more frequently presented with what was termed “atypical symptomatology” [92]. Therapeutically, it seemed that the limited medical care was similar regardless of sex [91]. The most pressing issue in Africa was delayed presentation impacting timely care [91,93] and access to state-of-the-art care. 

Hospital mortality from ACS is highly variable in Africa (1.2–24.5%), given varying study populations and the availability of revascularization procedures [9]. Limited sex-disaggregated data are available overall; while female sex was a predictor of long-term mortality in STEMI patients in Côte d’Ivoire [94], in the larger RACE-Nigeria registry, sex was not a predictor of mortality [89]. No sex differences in mortality were demonstrated in Senegal and Sudan [92,93]. 

There is a need to increase focus on sex-specific research and ACS research in general in Africa, particularly as most available registries are small, with an under-representation of women [9,89]. It is unclear if women experience ACS at lower rates than men or if women are less likely to access medical care. Existing large-scale registries such as the multinational ACCESS (Acute Coronary Events—A Multinational Survey of Current Management Strategies) registry [95] should also be leveraged to report sex-disaggregated data for ACS management patterns and outcomes. A framework to optimize care based on realistic considerations and available healthcare facilities in the region is needed, together with public awareness of timely presentation [10,93].

## 9. Eastern Mediterranean Region (EMR)

The EMR includes countries of the Arabian Gulf, Middle East and Northern Africa (MENA), and Western Asia. Large ACS registries and sex-specific analyses exist in the region, particularly from Saudi Arabia [18,20], the United Arab Emirates (UAE) [17,96,97], Egypt [98] and Pakistan [99]. 

Women constituted less than a quarter of patients across all major registries in MENA, including the Saudi Project for Assessment of Coronary Events (SPACE) [20], Gulf Registry of Acute Coronary Events (Gulf RACE) [17], and the Egyptian cross-sectional CardioRisk project [100]. Female patients were older, had more diabetes, hypertension, hyperlipidemia and high BMI [17,18,20,96,100], with fewer smokers [18], as in SSA, reflecting a sociocultural pattern. As with SSA, women from Northern Africa presented later, with longer ED stays, albeit similar median decision times to treatment [96,101]. 

NSTEMI was the more frequent ACS diagnosis among women [17,20,100,102]. In-hospital mortality rates were significantly higher in women compared with men for both STEMI and NSTEMI [17,20,96]. Women also had a greater prevalence of heart failure, atrial fibrillation and 1-year mortality [17,18,20].

Age-related disparities in ACS care have led to worse outcomes among younger women than younger men [96], and although younger women (aged < 65 years) with STEMI were more likely to seek acute medical care, they were less likely to undergo invasive diagnostic or therapeutic revascularization therapies (thrombolysis and primary PCI), or GDMT [17,96].

Recently, a shift from the recognized tendency to treat men more aggressively has been demonstrated. The Egyptian CardioRisk study found no gender differences in most diagnostic and therapeutic procedures, including primary PCI [98]. In the Saudi Acute Myocardial Infarction Registry Program (STARS-1 Program), implementation of GDMT did not differ based on sex, despite women being older with more CV risk factors [18]. Consequently, there were also no sex differences in in-hospital mortality [18].

Pakistan, a country generally grouped within South Asia, shares the South Asian CVD characteristics of the Indian subcontinent [103] but is categorized within the EMR by the WHO. Demographics were similar to EMR and South Asian registries [99], but women had longer pre-hospital delays in seeking treatment, attributing these delays to social factors, such as worry about expenses and bystanders’ responses to their symptoms. This differed from men, whose delays were attributed to not recognizing the symptoms as cardiac [104]. This reflects an underlying cultural aspect that might influence treatment-seeking behavior, where women are often curtailed in decision making and access to healthcare, owing to dependence on both finances and mobility [104]. Women were less aggressively treated with coronary interventions and had more adverse events as compared to males [99], although impaired renal dynamics were more frequently seen among men, similar to a large Bangladeshi ACS cohort [105].

## 10. South-East Asian Region (SEAR)

The SEAR is home to a large population, additionally predisposed to the South Asian phenotype of CAD. The South Asian nations of India, Pakistan, Bangladesh, Sri Lanka, and Nepal account for about a quarter of the world’s population and contribute the highest proportion of the world’s CVD burden, many of whom are also young [103]. There are considerable data on sex differences of ACS in the region, particularly from India [11,12,106,107,108] and Bangladesh [18,105,109,110], albeit women comprising less than a quarter of participants [11,12,105,106]. Demographics were similar to global patterns, with women being older, with more CV risk factors, notably more diabetes and hypertension [11,12,105,106,107,108,109,111,112]. Sex-based analyses of Indian ACS registries reveal conflicting evidence on ACS care [11,12,106,108]. The Detection and Management of Coronary Heart Disease (DEMAT) Registry and the large Kerala ACS registries both noted no between-sex differences in inpatient diagnostics, management and discharge care, with similar 30-day mortality, and composites of death, reinfarction, stroke, heart failure, cardiogenic shock or cardiac arrest [11,108]. In contrast, the more recent ACS QUIK trial (Acute Coronary Syndrome Quality Improvement in Kerala) from a similar region reported longer delays in hospital presentation for women and less likelihood of receiving primary PCI and GDMT, with worse in-hospital and 30-day major adverse cardiac events (MACE) [12]. This was similar to older data from the CREATE registry, wherein women had lower rates of revascularization, GDMT and higher unadjusted all-cause 30-day mortality [106,107].

In the largest ACS registry in Bangladesh, women were less likely to undergo coronary angiography and PCI, which did not translate into disparities in in-hospital outcomes, including mortality, except for the reduced risk of AKI as compared with men [105]. However, younger Bangladeshi women with ACS undergoing PCI experienced significantly greater short-term MACE, notably excess bleeding mainly related to access to the site, vascular complications and recurrent ischemia [110].

The large Acute Coronary Syndrome Sri Lanka Audit Project (ACSSLAP) found no sex disparities in management strategies or risk factor target achievements when stratified by age, except for better control of fasting blood sugar by men. Notably, in-hospital and 1-year mean mortality risk was significantly higher among men less than 65 years of age (*p* < 0.05) [111].

Among STEMI patients undergoing primary PCI in Thailand, despite no sex differences in door-to-balloon time, women experienced greater short and long-term mortality and were less likely to receive aspirin and statin at discharge [113]. A decade earlier, women in Thailand were less likely to undergo angiography, or PCI, or to receive thrombolysis, with higher mortality [112].

Data on sex disparities from other SEAR countries are sparse: a smaller pilot study of 53 ACS patients (47.2% women) in Indonesia documented differences in chest pain characteristics between men and women, with no data on sex differences in outcomes [114].

## 11. Western Pacific Region (WPR)

The Western Pacific region includes Oceania and far East Asia, comprising varying healthcare systems. Much of the region’s data come from large Australian registries where women were older, more likely to present with NSTE-ACS and have more comorbidities, with delays in presentation and revascularization [5,87,115]. In the large Australian Cooperative National Registry of Acute Coronary care, Guideline Adherence and Clinical Events (CONCORDANCE) registry, significantly more women had non-obstructive coronary artery disease (NOCAD) [115].

Australian women with ACS were less likely to receive interventions including angiograms, PCI and CABG [116], which persisted among NSTE-ACS patients even after adjusting for number of comorbidities [5,37], indicating a potential gender bias in referral of treatments [116]. In an ACS cohort with no baseline sex differences in comorbidities or coronary angiography, women with NSTEMI were 44% less likely to receive grafts [116]. Despite disparities in care, in the majority of Australian data, outcomes including adjusted in-hospital mortality and MACE rates were similar for men and women [5,87,115].

In the Malaysian NCVD-ACS (National Cardiovascular Disease Database-Acute Coronary Syndrome) registry, women had higher in-hospital mortality in STEMI but not NSTE-ACS [15]. Women were significantly less likely to receive both primary PCI and fibrinolysis [78]. Angiographically, women had significantly more left main and small vessel disease, with longer door-to-balloon in STEMI [78]. 

An age bias in presentation, revascularization modalities and outcomes exist. In older data from Singapore, younger women had worse 28-day survival rates and were more likely to present with “atypical” symptoms, have prior CAD and require resuscitation, none of which were observed among older women, indicating age-specific gender differences [117]. 

In China, where the burden of ACS is significant, differences in invasive coronary procedures were greater in younger individuals in contemporary registries, on the background of lower use of invasive angiography and acute reperfusion therapy, including heparin and dual antiplatelet therapy among women overall [79,118]. Younger Australian women (<50 years) were less likely to receive a stent, and older women (>50 years) were less likely to receive grafts [116]. Contrary to trends of worse outcomes among younger women, South Korean women > 65 years had more complications of ACS, underwent fewer invasive procedures, and ultimately had higher short-term mortality when compared with men or younger women [119].

A trend towards a differential impact of risk factors was also observed in the Japanese Acute Coronary Syndrome Study (JACSS): women had greater odds of AMI in the presence of smoking and diabetes than with hypertension and a family history of CAD [120]. Smoking as a risk factor for AMI was far more significant for women compared with men, with 37% of current smokers being women [120]. Although women had higher in-hospital mortality than men after primary PCI, the female sex itself was not an independent predictor of mortality [121].

## 12. Region of the Americas (AMR)

Sex differences in the presentation, management and outcomes of ACS across the Americas, especially in North America, have been particularly well-documented by leveraging large-scale registries [7,122,123], such as the American College of Cardiology-National Cardiovascular Data Registry (ACC-NCDR) [124], American Heart Association Get with The Guidelines Coronary Artery Disease Registry [125] and national in-patient sample databases [2]. The increase in the use of invasive cardiac procedures has led to a greater focus on coronary plaque morphology [20], etiology, bleeding [126,127] and outcomes following PCI, with dedicated research being borne from large-scale registries such as the NCDR [127].

A temporal analysis of a large US national inpatient sample of >7 million AMI patients over 12 years found that in addition to regular CV risk factors, women were also more likely to have chronic kidney disease (CKD), with depression and hypothyroidism also being substantially more prevalent [2]. These non-traditional risk factors are not commonly assessed in most regions of the world and address an unmet need in CVD research.

Despite advances in invasive management and pharmacotherapy, women were still less likely to receive angiography and PCI in the US, Canada, and South and Central American countries such as Brazil, Argentina and Mexico, with higher all-cause mortality and MACE [2,7,128,129,130]. A unique comparison of sex differences in STEMI patients in Sweden and Canada (Canadian Global Registry of Acute Coronary Events) found that female sex was associated with less use of reperfusion therapy in both countries, which persisted after multivariable adjustments including prehospital delay, atypical symptoms, and diabetes [131].

Sex differences in outcomes in North America have been largely attributed to the receipt and timing of invasive treatments, with a potential bias at play in care delivery. Despite there being no sex-specific guidelines on the timing of invasive care, women were less likely to experience guideline-mandated cardiac catheterization than men [1,81], particularly in STEMI [1]. Even during the COVID-19 pandemic, the use of PCI was less frequent and medical therapy more frequent among women with STEMI, who also had an identified culprit lesion less often than men [132]. Fewer women underwent an early invasive-treatment approach in NSTEACS, with higher age and renal insufficiency predictive of a conservative approach [133]. In large Canadian pooled analyses too, an under-use of invasive angiography and GDMT was seen for women with NSTEACS, who also suffered higher in-hospital mortality [7]. However, at least in the United States, higher STEMI mortality in women is largely attributed as being due to comorbidities and concomitant CV risk factors rather than treatment discrepancies [122,134]. Furthermore, lower referral to cardiac rehabilitation, more dropouts, and lower levels of physical activity among women contribute to overall poorer improvements in their health status post-MI [135].

Most sex-based differences in plaque burden and composition are observed in non-culprit lesions, at a younger age, with attenuation of differences over the age of 65 years [20]. Women also demonstrate greater visual-functional mismatch on Fractional flow reserve (FFR) evaluation compared with angiography and IVUS [20]. Higher rates of procedural complications, particularly vascular access site and bleeding complications, have been consistently reported in women [2,127].

Overall, evidence-based invasive and pharmacological therapies still remain paradoxically targeted toward low-risk patients, even in high-income countries [123,133,136]. Furthermore, an underestimation of patient risk has also been attributed to the non-pursuit of an invasive strategy in both men and women [7]. The challenge, thus, is to develop strategies to eliminate this treatment-risk paradox. 

## 13. European Region (EUR)

Similar to the Americas, large registries from Western Europe reflect excess mortality and persistent sex-based differences in ACS care among women, with less GDMT and invasive revascularization procedures [6,14,137,138,139,140]. Furthermore, reperfusion strategies in women less frequently involved radial access [4,6,14], thrombo-aspiration [14] and drug-eluting stent (DES) implantation [139]. Even among NSTEMI patients at the highest risk of ischemic complications, women were less likely to receive invasive management; even when they did receive an invasive strategy, consistent delays were observed compared with men [4]. Furthermore, despite older age and unfavorable risk profile, female ACS patients seem to be sub-optimally treated with P2Y12 inhibitors [139].

Women have less obstructive CAD [6,137,140], as demonstrated in the Swedish Coronary Angiography and Angioplasty Registry (SCAAR), where NOCAD in STEMI and NSTEMI was significantly greater among women [141]. Women have higher rates of MINOCA due to plaque erosion, coronary spasm, CMD and SCAD [142], and type II AMI [32]. 

Post-PCI, female sex was an independent predictor of all-cause mortality at 30 days and 1 year among ACS patients in a combined British Cardiovascular Intervention Society (BCIS) and SCAAR registry analysis [143]. Women were also at risk of access-site but not overall or non-access site bleeding, an association which remained consistent only for the femoral approach [144]. Paradoxically, fewer women underwent invasive procedures transradially [6,14].

In the International Registry of Acute Coronary Syndromes registry study in Transitional Countries (ISACS-TC), a long-term ACS registry in Central and Eastern Europe [145], an interesting treatment paradox was observed: women managed with routine medical care had more favorable outcomes as compared with men, whereas women undergoing PCI experienced higher rates adverse outcomes [146]. An age-sex paradox was also seen, where in-hospital mortality from ACS was not different between older men and women but higher amongst women < 65 years after STEMI when compared with similarly aged men [7,147]. Sex differences in STEMI mortality were driven by prehospital delays in presentation [148]. Women had a higher risk of de novo heart failure after STEMI, in whom worse survival was seen [149]. Among nonagenarians with ACS, women demonstrated better clinical outcomes post-PCI, despite receiving less invasive approaches [150], underscoring the importance of considering elderly women with NSTE-ACS for early revascularization [151]. 

Similar to trends in the Middle East, sex differences seem to be narrowing in Europe. In Poland, sex differences in STEMI patients reduced from 2005 to 2011 [152]. Likewise, there were no sex differences in time to angiography, revascularization rates, in-hospital and one-year mortality in recent Spanish data, although women were still less likely to receive potent antiplatelet agents [153]. 

## 14. Closing the Gaps

**Figure 2:**
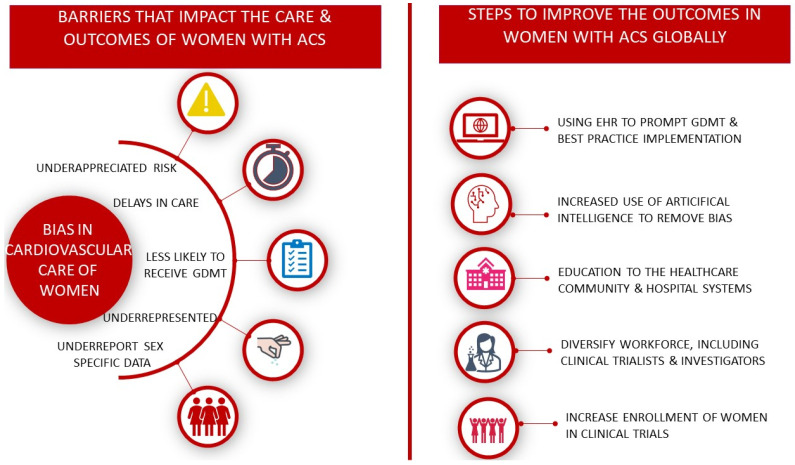
Bias in the care of women with acute coronary syndromes and actionable items to improve outcomes. *Guideline Directed medical therapy (GDMT)*.

Despite women being the majority of the population, they remain the “second sex” in terms of the medical community. Despite the fact that over three decades ago, Dr. Bernadine Healy called for the medical community to take women’s cardiac symptoms seriously and implemented a policy that women be included in National Institute of Health (NIH) funded cardiovascular trials [154], women remain underrepresented, sex-specific data often go unreported, women’s symptoms of angina are often underappreciated, and women with STEMI remain undertreated when compared with men [133] (Figure 2). This structural sex bias in cardiovascular care is ultimately killing women; in some parts of the world, it is deeply ingrained within social constructs and requires active community intervention to circumvent.

## 15. Underrepresentation of Women in Clinical Trials

Further affecting the ability to optimally treat women is the lack of enrolment of women in cardiovascular clinical trials. For ACS and CAD, clinical trials from 2010 to 2017 continue to enroll fewer women than men, with a median female-to-male ratio of 0.34, with persistent gaps after adjusting for the prevalence of the disease by sex [155]. These sex gaps in inclusion are seen in drug trials, device and intervention trials, and lifestyle interventions [155]. The persistent underrepresentation of women in trials contributes to our lack of understanding of sex differences in treatments of STEMI and the inability to identify therapies or interventions that may uniquely benefit women.

## 16. Targeted Action Plans

Steps towards improving the care of women who present with STEMI require efforts directed at patients, the healthcare community and targeted, inclusive research [76]. Increasing public awareness of risk in women is a priority in order to ensure timely care for potential cardiac symptoms. It is well-documented that women are both unaware of their risk and underestimate their risk for CVD [156]. Additionally, until recently, the public health message related to symptoms of ACS in women has focused on the idea that women experience “atypical” symptoms. Increasing the awareness of symptoms that suggest a MI and the need for timely evaluation and treatment should be prioritized for women. Such efforts have been initiated by the American Heart Association’s “Go Red for Women” campaign [157], but despite these efforts, awareness has declined in women in the past decade, particularly in women under the age of 65 years [156].

Nonetheless, the bias in care requires efforts directed toward the medical community. Education and bias mitigation training are required to overcome the persistent differences in the treatment of ACS in women compared with men [158,159]. In regions where technology permits, additional improvements in ACS outcomes of women can be recognized by the use of protocol-driven care of STEMI to further reduce bias based on sex or race, using prompts through electronic health records and ensuring adherence to timely and guideline-directed medical therapies [76,85]. Furthermore, incorporating artificial intelligence into the assessment and care of patients with suspected ACS may assist in narrowing the gaps in care for all patients, irrespective of sex [160]. Finally, further research in the management of STEMI/ACS must make greater efforts to include women in order to understand the underlying pathophysiology, medical and interventional therapies, and identify sex differences where they exist [80].

## 17. Conclusions

Evidence of sex-related disparities in ACS prognosis first emerged over 30 years ago from the Framingham study [161]. There remains a disparity not only in sex differences across the different regions internationally but also in the specific issues that need to be tackled to overcome these differences in each specific region, considering the availability of resources, local practice patterns and access to care. Particularly in Asia and Africa, other factors, including ethnicity, culture, psychosocial, educational and socioeconomic statuses, might contribute to the sex differences in ACS. The longstanding knowledge gap and understanding of CAD in women, both on the part of physicians and of patients, has led to bias, which is at least in part has been responsible for the inequalities in healthcare access and processes. While this has been somewhat mitigated with advanced research and training in the Americas and Europe, there is an urgent need for more data in regions where they are lacking so that both patients and physicians alike and adequately informed and better prepared for reducing sex disparities in care and outcomes following ACS.

## Data Availability

Not applicable.

## Appendix A. List of World Health Organization (WHO) Regions and Countries Belong to Each Region

**African Region (AFR)**

Algeria, Angola, Benin, Botswana, Burkina Faso, Burundi, Cameroon, Cape Verde, Central African Republic, Chad, Comoros, Ivory Coast, Democratic Republic of the Congo, Equatorial Guinea, Eritrea, Ethiopia, Gabon, Gambia, Ghana, Guinea, Guinea-Bissau, Kenya, Lesotho, Liberia, Madagascar, Malawi, Mali, Mauritania, Mauritius, Mozambique, Namibia, Niger, Nigeria, Republic of the Congo, Rwanda, São Tomé and Príncipe, Senegal, Seychelles, Sierra Leone, South Africa, South Sudan, Eswatini, Togo, Uganda, Tanzania, Zambia, Zimbabwe

**Region of the Americas (AMR)**

Antigua and Barbuda, Argentina, Bahamas, Barbados, Belize, Bolivia, Brazil, Canada, Chile, Colombia, Costa Rica, Cuba, Dominica, Dominican Republic, Ecuador, El Salvador, Grenada, Guatemala, Guyana, Haiti, Honduras, Jamaica, Mexico, Nicaragua, Panama, Paraguay, Peru, Saint Kitts and Nevis, Saint Lucia, Saint Vincent and the Grenadines, Suriname, Trinidad and Tobago, United States, Uruguay, Venezuela.

**South-East Asian Region (SEAR)**

Bangladesh, Bhutan, North Korea, India, Indonesia, Maldives, Myanmar, Nepal, Sri Lanka, Thailand, Timor-Leste.

**European Region (EUR)**

Albania, Andorra, Armenia, Austria, Azerbaijan, Belarus, Belgium, Bosnia and Herzegovina, Bulgaria, Croatia, Cyprus, Czech Republic, Denmark, Estonia, Finland, France, Georgia, Germany, Greece, Hungary, Iceland, Ireland, Israel, Italy, Kazakhstan, Kyrgyzstan, Latvia, Lithuania, Luxembourg, Malta, Moldova, Monaco, Montenegro, Netherlands, North Macedonia, Norway, Poland, Portugal, Romania, Russia, San Marino, Serbia, Slovakia, Slovenia, Spain, Sweden, Switzerland, Tajikistan, Turkey, Turkmenistan, Ukraine, United Kingdom, Uzbekistan.

**Eastern Mediterranean Region (EMR)**

Afghanistan, Bahrain, Djibouti, Egypt, Iran, Iraq, Jordan, Kuwait, Lebanon, Libya, Morocco, Oman, Pakistan, Palestine, Qatar, Saudi Arabia, Somalia, Sudan, Syria, Tunisia, United Arab Emirates, Yemen.

**Western Pacific Region (WPR)**

Australia, Brunei, Cambodia, China, Cook Islands, Fiji, Japan, Kiribati, Laos, Malaysia, Marshall Islands, Micronesia, Mongolia, Nauru, New Zealand, Niue, Palau, Papua New Guinea, Philippines, Samoa, Singapore, Solomon Islands, South Korea, Tonga, Tuvalu, Vanuatu, Vietnam.

## References

[1] Collet J.P., Thiele H., Barbato E., Barthélémy O., Bauersachs J., Bhatt D.L., Dendale P., Dorobantu M., Edvardsen T., Folliguet T. (2021). 2020 ESC Guidelines for the management of acute coronary syndromes in patients presenting without persistent ST-segment elevation The Task Force for the management of acute coronary syndromes in patients presenting without persistent ST-segment elevation of the European Society of Cardiology (ESC). Eur. Heart J..

[2] Jneid H., Fonarow G.C., Cannon C.P., Hernandez A.F., Palacios I.F., Maree A.O., Maree A.O., Wells Q., Bozkurt B., Labresh K.A. (2008). Sex differences in medical care and early death after acute myocardial infarction. Circulation.

[3] Matetic A., Shamkhani W., Rashid M., Volgman A.S., Van Spall H.G.C., Coutinho T., Mehta L.S., Sharma G., Parwani P., Mohamed M.O. (2021). Trends of Sex Differences in Clinical Outcomes After Myocardial Infarction in the United States. CJC Open.

[4] Rashid M., Curzen N., Kinnaird T., Lawson C.A., Myint P.K., Kontopantelis E., Mohamed M.O., Shoaib A., Gale C.P., Timmis A. (2020). Baseline risk, timing of invasive strategy and guideline compliance in NSTEMI: Nationwide analysis from MINAP. Int. J. Cardiol..

[5] Worrall-Carter L., McEvedy S., Wilson A., Rahman M.A. (2016). Gender Differences in Presentation, Coronary Intervention, and Outcomes of 28,985 Acute Coronary Syndrome Patients in Victoria, Australia. Women’s Health Issues.

[6] ten Haaf M.E., Bax M., ten Berg J.M., Brouwer J., van’t Hof A.W., van der Schaaf R.J., Stella P.R., Tjon Joe Gin R.M., Tonino P.A., de Vries A.G. (2019). Sex differences in characteristics and outcome in acute coronary syndrome patients in the Netherlands. Neth. Heart J..

[7] Cenko E., Yoon J., Kedev S., Stankovic G., Vasiljevic Z., Krljanac G., Kalpak O., Ricci B., Milicic D., Manfrini O. (2018). Sex Differences in Outcomes After STEMI: Effect Modification by Treatment Strategy and Age. JAMA Intern. Med..

[8] Poon S., Goodman S.G., Yan R.T., Bugiardini R., Bierman A.S., Eagle K.A., Johnston N., Huynh T., Grondin F.R., Schenck-Gustafsson K. (2012). Bridging the gender gap: Insights from a contemporary analysis of sex-related differences in the treatment and outcomes of patients with acute coronary syndromes. Am. Heart J..

[9] Yao H., Ekou A., Niamkey T., Gan S.H., Kouamé I., Afassinou Y., Ehouman E., Touré C., Zeller M., Cottin Y. (2022). Acute Coronary Syndromes in Sub-Saharan Africa: A 10-Year Systematic Review. J. Am. Heart Assoc..

[10] Yuyun M.F., Sliwa K., Kengne A.P., Mocumbi A.O., Bukhman G. (2020). Cardiovascular Diseases in Sub-Saharan Africa Compared to High-Income Countries: An Epidemiological Perspective. Glob. Heart.

[11] Tsao C.W., Aday A.W., Almarzooq Z.I., Alonso A., Beaton A.Z., Bittencourt M.S., Boehme A.K., Buxton A.E., Carson A.P., Commodore-Mensah Y. (2022). Heart Disease and Stroke Statistics—2022 Update: A Report from the American Heart Association. Circulation.

[12] Pagidipati N.J., Huffman M.D., Jeemon P., Gupta R., Negi P., Jaison T.M., Sharma S., Sinha N., Mohanan P., Muralidhara B.G. (2013). Association between Gender, Process of Care Measures, and Outcomes in ACS in India: Results from the Detection and Management of Coronary Heart Disease (DEMAT) Registry. PLoS ONE.

[13] Khraishah H., Alahmad B., Alfaddagh A., Jeong S.Y., Mathenge N., Kassab M.B., Kolte D., Michos E.D., Albaghdadi M. (2021). Sex disparities in the presentation, management and outcomes of patients with acute coronary syndrome: Insights from the ACS QUIK trial. Open Heart.

[14] Leurent G., Garlantézec R., Auffret V., Hacot J.P., Coudert I., Filippi E., Rialan A., Moquet B., Rouault G., Gilard M. (2014). Gender differences in presentation, management and inhospital outcome in patients with ST-segment elevation myocardial infarction: Data from 5000 patients included in the ORBI prospective French regional registry. Arch. Cardiovasc. Dis..

[15] Lu H.T., Nordin R., Ahmad W.A.W., Lee C.Y., Zambahari R., Ismail O., Liew H.B., Sim K.H. (2014). NCVD Investigators Sex differences in acute coronary syndrome in a multiethnic asian population: Results of the malaysian national cardiovascular disease database-acute coronary syndrome (NCVD-ACS) registry. Glob. Heart.

[16] Bugiardini R., Badimon L., ISACS-TC Investigators and Coordinators (2016). The International Survey of Acute Coronary Syndromes in Transitional Countries (ISACS-TC): 2010–2015. Int. J. Cardiol..

[17] Shehab A., Al-Dabbagh B., AlHabib K.F., Alsheikh-Ali A.A., Almahmeed W., Sulaiman K., Al-Motarreb A., Nagelkerke N., Al Suwaidi J., Hersi A. (2013). Gender Disparities in the Presentation, Management and Outcomes of Acute Coronary Syndrome Patients: Data from the 2nd Gulf Registry of Acute Coronary Events (Gulf RACE-2). PLoS ONE.

[18] Kinsara A.J., Ismail Y.M. (2021). Gender differences in patients presenting with non-ST segment elevation myocardial infarction in the STAR registry. Egypt. Heart J. Off. Bull. Egypt. Soc. Cardiol..

[19] Cader F.A., Haq M.M., Nasrin S., Kabir C.S. (2017). Presentation, Management Practices and In-hospital Outcomes of Patients with Acute Coronary Syndrome in a Tertiary Cardiac Centre in Bangladesh. Bangladesh Heart J..

[20] Hersi A., Al-Habib K., Al-Faleh H., Al-Nemer K., AlSaif S., Taraben A., Kashour T., Abuosa A.M., Al-Murayeh M.A. (2013). Gender inequality in the clinical outcomes of equally treated acute coronary syndrome patients in Saudi Arabia. Ann. Saudi Med..

[21] Chandrasekhar J., Mehran R. (2016). Sex-Based Differences in Acute Coronary Syndromes: Insights from Invasive and Noninvasive Coronary Technologies. JACC Cardiovasc. Imaging.

[22] Regitz-Zagrosek V., Oertelt-Prigione S., Prescott E., Franconi F., Gerdts E., Foryst-Ludwig A., Maas A.H., Kautzky-Willer A., Knappe-Wegner D., Kintscher U. (2016). Gender in cardiovascular diseases: Impact on clinical manifestations, management, and outcomes. Eur. Heart J..

[23] Stähli B.E., Gebhard C., Yonekawa K., Gebhard C.E., Altwegg L.A., Von Eckardstein A., Hersberger M., Novopashenny I., Wolters R., Wischnewsky M.B. (2015). Gender-Related Differences in Patients Presenting with Suspected Acute Coronary Syndromes: Clinical Presentation, Biomarkers and Diagnosis. Cardiology.

[24] Johansson I., Strömberg A., Swahn E. (2004). Factors related to delay times in patients with suspected acute myocardial infarction. Heart Lung.

[25] Diercks D.B., Owen K.P., Kontos M.C., Blomkalns A., Chen A.Y., Miller C., Wibiott S., Peterson E.D. (2010). Gender differences in time to presentation for myocardial infarction before and after a national women’s cardiovascular awareness campaign: A temporal analysis from the Can Rapid Risk Stratification of Unstable Angina Patients Suppress ADverse Outcomes with Early Implementation (CRUSADE) and the National Cardiovascular Data Registry Acute Coronary Treatment and Intervention Outcomes Network-Get with the Guidelines (NCDR ACTION Registry-GWTG). Am. Heart J..

[26] Thuresson M., Jarlöv M.B., Lindahl B., Svensson L., Zedigh C., Herlitz J. (2005). Symptoms and type of symptom onset in acute coronary syndrome in relation to ST elevation, sex, age, and a history of diabetes. Am. Heart J..

[27] Canto J.G., Goldberg R.J., Hand M.M., Bonow R.O., Sopko G., Pepine C.J., Long T. (2007). Symptom presentation of women with acute coronary syndromes: Myth vs reality. Arch. Intern. Med..

[28] McSweeney J.C., Cleves M.A., Fischer E.P., Rojo M.O., Armbya N., Moser D.K. (2013). Reliability of the McSweeney Acute and Prodromal Myocardial Infarction Symptom Survey among black and white women. Eur. J. Cardiovasc. Nurs..

[29] Wouters L.T.C.M., Zwart D.L.M., Erkelens D.C.A., De Groot E., Van Smeden M., Hoes A.W., Damoiseaux R.A.M.J., Rutten F.H. (2021). Gender-stratified analyses of symptoms associated with acute coronary syndrome in telephone triage: A cross-sectional study. BMJ Open.

[30] Khan N.A., Daskalopoulou S.S., Karp I., Eisenberg M.J., Pelletier R., Tsadok M.A., Dasgupta K., Norris C.M., Pilote L., GENESIS PRAXY Team (2013). Sex differences in acute coronary syndrome symptom presentation in young patients. JAMA Intern. Med..

[31] Ferry A.V., Anand A., Strachan F.E., Mooney L., Stewart S.D., Marshall L., Chapman A.R., Lee K.K., Jones S., Orme K. (2019). Presenting Symptoms in Men and Women Diagnosed with Myocardial Infarction Using Sex-Specific Criteria. J. Am. Heart Assoc..

[32] Lichtman J.H., Leifheit E.C., Safdar B., Bao H., Krumholz H.M., Lorenze N.P., Daneshvar M., Spertus J.A., D’Onofrio G. (2018). Sex differences in the presentation and perception of symptoms among young patients with myocardial infarction. Circulation.

[33] Gulati M., Levy P.D., Mukherjee D., Amsterdam E., Bhatt D.L., Birtcher K.K., Blankstein R., Boyd J., Bullock-Palmer R.P., Writing Committee Members (2021). 2021 AHA/ACC/ASE/CHEST/SAEM/SCCT/SCMR Guideline for the Evaluation and Diagnosis of Chest Pain: Executive Summary: A Report of the American College of Cardiology/American Heart Association Joint Committee on Clinical Practice Guidelines. J. Am. Coll. Cardiol..

[34] Elder P., Sharma G., Gulati M., Michos E.D. (2020). Identification of female-specific risk enhancers throughout the lifespan of women to improve cardiovascular disease prevention. Am. J. Prev. Cardiol..

[35] The Global Use of Strategies to Open Occluded Coronary Arteries in Acute Coronary Syndromes (GUSTO IIb) Angioplasty Substudy Investigators (1997). A Clinical Trial Comparing Primary Coronary Angioplasty with Tissue Plasminogen Activator for Acute Myocardial Infarction. N. Engl. J. Med..

[36] Agarwal A., Doshi S. (2013). The role of oxidative stress in menopause. J. Midlife Health.

[37] Worrall-Carter L., McEvedy S., Wilson A., Rahman M.A. (2016). Impact of comorbidities and gender on the use of coronary interventions in patients with high-risk non-ST-segment elevation acute coronary syndrome. Catheter. Cardiovasc. Interv..

[38] Dreyer R.P., Smolderen K.G., Strait K.M., Beltrame J.F., Lichtman J.H., Lorenze N.P., D’Onofrio G., Bueno H., Krumholz H.M., Spertus J.A. (2016). Gender differences in pre-event health status of young patients with acute myocardial infarction: A VIRGO study analysis. Eur. Heart J. Acute Cardiovasc. Care.

[39] Yusuf P.S., Hawken S., Ôunpuu S., Dans T., Avezum A., Lanas F., McQueen M., Budaj A., Pais P., Varigos J. (2004). Effect of potentially modifiable risk factors associated with myocardial infarction in 52 countries (the Interheart study): Case-control study. Lancet.

[40] Njølstad I., Arnesen E., Lund-Larsen P.G. (1996). Smoking, serum lipids, blood pressure, and sex differences in myocardial infarction: A 12-year follow-up of the Finnmark Study. Circulation.

[41] Banerjee A., Silver L.E., Heneghan C., Welch S.J.V., Bull L.M., Mehta Z., Banning A.P., Rothwell P.M. (2009). Sex-specific familial clustering of myocardial infarction in patients with acute coronary syndromes. Circ. Cardiovasc. Genet..

[42] Bucholz E.M., Strait K.M., Dreyer R.P., Lindau S.T., D’Onofrio G., Geda M., Spatz E.S., Beltrame J.F., Lichtman J.H., Lorenze N.P. (2017). Sex Differences in Young Patients with Acute Myocardial Infarction: A VIRGO Study Analysis. Eur. Heart J. Acute Cardiovasc. Care.

[43] Backholer K., Peters S.A.E., Bots S.H., Peeters A., Huxley R.R., Woodward M. (2017). Sex differences in the relationship between socioeconomic status and cardiovascular disease: A systematic review and meta-analysis. J. Epidemiol. Community Health.

[44] Forrester S., Jacobs D., Zmora R., Schreiner P., Roger V., Kiefe C.I. (2019). Racial differences in weathering and its associations with psychosocial stress: The CARDIA study. SSM Popul. Health.

[45] Grundy S.M., Stone N.J., Bailey A.L., Beam C., Birtcher K.K., Blumenthal R.S., Braun L.T., de Ferranti S., Faiella-Tommasino J., Forman D.E. (2019). 2018 AHA/ACC/AACVPR/AAPA/ABC/ACPM/ADA/AGS/APhA/ASPC/NLA/PCNA Guideline on the Management of Blood Cholesterol: A Report of the American College of Cardiology/American Heart Association Task Force on Clinical Practice Guidelines. Circulation.

[46] Wu P., Park K., Gulati M. (2021). The fourth trimester: Pregnancy as a predictor of cardiovascular disease. Eur. Cardiol. Rev..

[47] Grandi S.M., Filion K.B., Yoon S., Ayele H.T., Doyle C.M., Hutcheon J.A., Smith G.N., Gore G.C., Ray J.G., Nerenberg K. (2019). Cardiovascular Disease-Related Morbidity and Mortality in Women with a History of Pregnancy Complications. Circulation.

[48] Tanz L.J., Stuart J.J., Williams P.L., Rimm E.B., Missmer S.A., Rexrode K.M., Mukamal K.J., Rich-Edwards J.W. (2017). Preterm Delivery and Maternal Cardiovascular Disease in Young and Middle-Aged Adult Women. Circulation.

[49] Kramer C.K., Campbell S., Retnakaran R. (2019). Gestational diabetes and the risk of cardiovascular disease in women: A systematic review and meta-analysis. Diabetologia.

[50] McKenzie-Sampson S., Paradis G., Healy-Profitós J., St-Pierre F., Auger N. (2018). Gestational diabetes and risk of cardiovascular disease up to 25 years after pregnancy: A retrospective cohort study. Acta Diabetol..

[51] Markovitz A.R., Stuart J.J., Horn J., Williams P.L., Rimm E.B., Missmer S.A., Tanz L.J., Haug E.B., Fraser A., Timpka S. (2019). Does pregnancy complication history improve cardiovascular disease risk prediction? Findings from the HUNT study in Norway. Eur. Heart J..

[52] Honigberg M.C., Zekavat S.M., Niroula A., Griffin G.K., Bick A.G., Pirruccello J.P., Nakao T., Whitsel E.A., Farland L.V., Laurie C. (2021). Premature Menopause, Clonal Hematopoiesis, and Coronary Artery Disease in Postmenopausal Women. Circulation.

[53] Osibogun O., Ogunmoroti O., Michos E.D. (2020). Polycystic ovary syndrome and cardiometabolic risk: Opportunities for cardiovascular disease prevention. Trends Cardiovasc. Med..

[54] Zhao L., Zhu Z., Lou H., Zhu G., Huang W., Zhang S., Liu F. (2016). Polycystic ovary syndrome (PCOS) and the risk of coronary heart disease (CHD): A meta-analysis. Oncotarget.

[55] Hiteshi A.K., Li D., Gao Y., Chen A., Flores F., Mao S.S., Budoff M.J. (2014). Gender differences in coronary artery diameter are not related to body habitus or left ventricular mass. Clin. Cardiol..

[56] Murthy V.L., Naya M., Taqueti V.R., Foster C.R., Gaber M., Hainer J., Dorbala S., Blankstein R., Rimoldi O., Camici P.G. (2014). Effects of sex on coronary microvascular dysfunction and cardiac outcomes. Circulation.

[57] Patel M.B., Bui L.P., Kirkeeide R.L., Gould K.L. (2016). Imaging Microvascular Dysfunction and Mechanisms for Female-Male Differences in CAD. JACC Cardiovasc. Imaging.

[58] Ong P., Camici P.G., Beltrame J.F., Crea F., Shimokawa H., Sechtem U., Kaski J.C., Bairey Merz C.N., Coronary Vasomotion Disorders International Study Group (COVADIS) (2018). International standardization of diagnostic criteria for microvascular angina. Int. J. Cardiol..

[59] Scalone G., Niccoli G., Crea F. (2019). Editor’s Choice- Pathophysiology, diagnosis and management of MINOCA: An update. Eur. Heart J. Acute Cardiovasc. Care.

[60] Pelliccia F., Pepine C.J., Berry C., Camici P.G. (2021). The role of a comprehensive two-step diagnostic evaluation to unravel the pathophysiology of MINOCA: A review. Int. J. Cardiol..

[61] Ouldzein H., Elbaz M., Roncalli J., Cagnac R., Carrié D., Puel J., Alibelli-Chemarin M.J. (2012). Plaque rupture and morphological characteristics of the culprit lesion in acute coronary syndromes without significant angiographic lesion: Analysis by intravascular ultrasound. Ann. Cardiol. Angeiol..

[62] Reynolds H.R., Srichai M.B., Iqbal S.N., Slater J.N., Mancini G.B.J., Feit F., Pena-Sing I., Axel L., Attubato M.J., Yatskar L. (2011). Mechanisms of myocardial infarction in women without angiographically obstructive coronary artery disease. Circulation.

[63] White S.J., Newby A.C., Johnson T.W. (2016). Endothelial erosion of plaques as a substrate for coronary thrombosis. Thromb. Haemost..

[64] Perera D., Berry C., Hoole S.P., Sinha A., Rahman H., Morris P.D., Kharbanda R.K., Petraco R., Channon K. (2022). Invasive coronary physiology in patients with angina and non-obstructive coronary artery disease: A consensus document from the coronary microvascular dysfunction workstream of the British Heart Foundation/National Institute for Health Research Partnership. Heart.

[65] Nishiguchi T., Tanaka A., Ozaki Y., Taruya A., Fukuda S., Taguchi H., Iwaguro T., Ueno S., Okumoto Y., Akasaka T. (2016). Prevalence of spontaneous coronary artery dissection in patients with acute coronary syndrome. Eur. Heart J. Acute Cardiovasc. Care.

[66] Saw J., Aymong E., Sedlak T., Buller C.E., Starovoytov A., Ricci D., Robinson S., Vuurmans T., Gao M., Humphries K. (2014). Spontaneous coronary artery dissection: Association with predisposing arteriopathies and precipitating stressors and cardiovascular outcomes. Circ. Cardiovasc. Interv..

[67] Tweet M.S., Hayes S.N., Pitta S.R., Simari R.D., Lerman A., Lennon R.J., Gersh B.J., Khambatta S., Best P.J., Rihal C.S. (2012). Clinical features, management, and prognosis of spontaneous coronary artery dissection. Circulation.

[68] Pasupathy S., Air T., Dreyer R.P., Tavella R., Beltrame J.F. (2015). Systematic review of patients presenting with suspected myocardial infarction and nonobstructive coronary arteries. Circulation.

[69] Crump R., Shandling A.H., Van Natta B., Ellestad M. (2000). Prevalence of patent foramen ovale in patients with acute myocardial infarction and angiographically normal coronary arteries. Am. J. Cardiol..

[70] Sastry S., Riding G., Morris J., Taberner D., Cherry N., Heagerty A., McCollum C. (2006). Young Adult Myocardial Infarction and Ischemic Stroke: The role of paradoxical embolism and thrombophilia (The YAMIS Study). J. Am. Coll. Cardiol..

[71] Sykes R., Doherty D., Mangion K., Morrow A., Berry C. (2021). What an interventionalist needs to know about MI with non-obstructive coronary arteries. Interv. Cardiol. Rev..

[72] Thygesen K., Alpert J.S., Jaffe A.S., Chaitman B.R., Bax J.J., Morrow D.A., White H.D., Executive Group on behalf of the Joint European Society of Cardiology (ESC)/American College of Cardiology (ACC)/American Heart Association (AHA)/World Heart Federation (WHF) Task Force for the Universal Definition of Myocardial Infarction (2018). Fourth Universal Definition of Myocardial Infarction (2018). Circulation.

[73] Tamis-Holland J.E., Jneid H., Reynolds H.R., Agewall S., Brilakis E.S., Brown T.M., Lerman A., Cushman M., Kumbhani D.J., Arslanian-Engoren C. (2019). Contemporary Diagnosis and Management of Patients with Myocardial Infarction in the Absence of Obstructive Coronary Artery Disease: A Scientific Statement from the American Heart Association. Circulation.

[74] Ghadri J.R., Wittstein I.S., Prasad A., Sharkey S., Dote K., Akashi Y.J., Cammann V.L., Crea F., Galiuto L., Desmet W. (2018). International Expert Consensus Document on Takotsubo Syndrome (Part I): Clinical Characteristics, Diagnostic Criteria, and Pathophysiology. Eur. Heart J..

[75] Galiuto L., De Caterina A.R., Porfidia A., Paraggio L., Barchetta S., Locorotondo G., Rebuzzi A.G., Crea F. (2010). Reversible coronary microvascular dysfunction: A common pathogenetic mechanism in Apical Ballooning or Tako-Tsubo Syndrome. Eur. Heart J..

[76] Gulati M. (2019). Yentl’s Bikini: Sex Differences in STEMI. J. Am. Heart Assoc..

[77] Sulaiman S., Kawsara A., Mohamed M.O., Van Spall H.G.C., Sutton N., Holmes D.R., Mamas M.A., Alkhouli M. (2021). Treatment Effect of Percutaneous Coronary Intervention in Men Versus Women With ST-Segment-Elevation Myocardial Infarction. J. Am. Heart Assoc..

[78] Lee C.Y., Ting K.L., Lu H.T., Ali R.M., Fong A.Y.Y., Wan Ahmad W.A. (2021). Sex and gender differences in presentation, treatment and outcomes in acute coronary syndrome, a 10 year study from a multi-ethnic Asian population: The Malaysian National Cardiovascular Disease Database-Acute Coronary Syndrome (NCVD-ACS) registry. PLoS ONE.

[79] Hao Y., Liu J., Liu J., Yang N., Smith S.C., Huo Y., Fonarow G.C., Ge J., Taubert K.A., Morgan L. (2019). Sex Differences in In-Hospital Management and Outcomes of Patients with Acute Coronary Syndrome. Circulation.

[80] Edmund Anstey D., Li S., Thomas L., Wang T.Y., Wiviott S.D. (2016). Race and Sex Differences in Management and Outcomes of Patients After ST-Elevation and Non-ST-Elevation Myocardial Infarct: Results From the NCDR. Clin. Cardiol..

[81] Udell J.A., Fonarow G.C., Maddox T.M., Cannon C.P., Frank Peacock W., Laskey W.K., Grau-Sepulveda M.V., Smith E.E., Hernandez A.F., Peterson E.D. (2018). Sustained sex-based treatment differences in acute coronary syndrome care: Insights from the American Heart Association Get with The Guidelines Coronary Artery Disease Registry. Clin. Cardiol..

[82] Peters S.A.E., Colantonio L.D., Zhao H., Bittner V., Dai Y., Farkouh M.E., Monda K.L., Safford M.M., Muntner P., Woodward M. (2018). Sex Differences in High-Intensity Statin Use Following Myocardial Infarction in the United States. J. Am. Coll. Cardiol..

[83] D’Onofrio G., Safdar B., Lichtman J.H., Strait K.M., Dreyer R.P., Geda M., Spertus J.A., Krumholz H.M. (2015). Sex differences in reperfusion in young patients with ST-segment-elevation myocardial infarction: Results from the VIRGO study. Circulation.

[84] Arora S., Stouffer G.A., Kucharska-Newton A.M., Qamar A., Vaduganathan M., Pandey A., Porterfield D., Blankstein R., Rosamond W.D., Bhatt D.L. (2019). Twenty Year Trends and Sex Differences in Young Adults Hospitalized With Acute Myocardial Infarction. Circulation.

[85] Huded C.P., Kumar A., Johnson M., Abdallah M., Ballout J.A., Kravitz K., Menon V., Gullett T.C., Hantz S., Ellis S.G. (2019). Incremental prognostic value of guideline-directed medical therapy, transradial access, and door-to-balloon time on outcomes in ST-segment-elevation myocardial infarction. Circ. Cardiovasc. Interv..

[86] Shah T., Haimi I., Yang Y., Gaston S., Taoutel R., Mehta S., Lee H.J., Zambahari R., Baumbach A., Henry T.D. (2021). Meta-Analysis of Gender Disparities in In-hospital Care and Outcomes in Patients with ST-Segment Elevation Myocardial Infarction. Am. J. Cardiol..

[87] Stehli J., Martin C., Brennan A., Dinh D.T., Lefkovits J., Zaman S. (2019). Sex Differences Persist in Time to Presentation, Revascularization, and Mortality in Myocardial Infarction Treated with Percutaneous Coronary Intervention. J. Am. Heart Assoc..

[88] WHO Alphabetical List of WHO Member States. https://www.who.int/countries.

[89] Isezuo S., Sani M.U., Talle A., Johnson A., Adeoye A.M., Ulgen M.S., Mbakwem A., Ogah O., Edafe E., Kolo P. (2022). Registry for Acute Coronary Events in Nigeria (RACE-Nigeria): Clinical Characterization, Management, and Outcome. J. Am. Heart Assoc. Cardiovasc. Cerebrovasc. Dis..

[90] Maurin O., Massoure P.L., De R.S., Topin F., Sbardella F., Lamblin G., Kaiser E. (2012). Acute myocardial infarction in Djibouti: 2-year prospective study. Med. Sante Trop..

[91] Mboup M.C., Mingou J., Ba D.M., Dia K., Fall P.D. (2019). Characteristics of acute coronary syndromes in Sub-Saharan African women. Ann. Cardiol. Angeiol..

[92] Marie D., Mingou J.S., Dia K., Gbadamassi S.E.O.K., Fall P.D., Diao M., Mboup M.C. (2019). Clinical Presentation, Risk Factor, and Outcomes of Acute Coronary Syndrome in Women at an Urban Referral Center in Dakar, Senegal. Glob. Heart.

[93] Mirghani H.O., Elnour M.A., Taha A.M., Elbadawi A.S. (2016). Gender inequality in acute coronary syndrome patients at Omdurman Teaching Hospital, Sudan. J. Family Community Med..

[94] Yao H., Ekou A., Hadéou A., N’Djessan J.J., Kouamé I., N’Guetta R. (2019). Medium and long-term follow-up after ST-segment elevation myocardial infarction in a sub-Saharan Africa population: A prospective cohort study. BMC Cardiovasc. Disord..

[95] Bazzino O., Monaco R., Mario B., Sergio C., Valeria C.M., Sergio E., Ricardo E., Juan G., Ernesto J., Carlos K. (2011). Management of acute coronary syndromes in developing countries: Acute coronary events-a multinational survey of current management strategies. Am. Heart J..

[96] Shehab A., Bhagavathula A.S., Alhabib K.F., Ullah A., Suwaidi J.A., Almahmeed W., AlFaleh H., Zubaid M. (2020). Age-Related Sex Differences in Clinical Presentation, Management, and Outcomes in ST-Segment–Elevation Myocardial Infarction: Pooled Analysis of 15 532 Patients From 7 Arabian Gulf Registries. J. Am. Heart Assoc. Cardiovasc. Cerebrovasc. Dis..

[97] El-Menyar A., Zubaid M., Sulaiman K., AlMahmeed W., Singh R., Alsheikh-Ali A.A., Al Suwaidi J., Gulf Registry of Acute Coronary Events (Gulf RACE) Investigators (2011). Atypical presentation of acute coronary syndrome: A significant independent predictor of in-hospital mortality. J. Cardiol..

[98] Reda A., Ashraf M., Soliman M., Ragy H., Elkersh A., Abdou W., Mostafa T., Hassan M., Farag E., Khamis H. (2018). Gender-related Differences in Risk factors and Treatment Strategies in Patients with Acute Coronary Syndrome across Egypt: Part of the Cardio- Risk Project. Atheroscler. Suppl..

[99] Altaf A., Shah H., Salahuddin M. (2019). Gender based differences in clinical and Angiographic characteristics and outcomes of Acute Coronary Syndrome (ACS) in Asian population. Pakistan J. Med. Sci..

[100] Reda A., Soliman M., El Kersh A., Abdou W., Mostafa M., Beshay M., Gamal A., Farag E., Mostafa T., El-Ghany M.A. (2019). The pattern of risk-factor profile in Egyptian patients with acute coronary syndrome: Phase II of the Egyptian cross-sectional CardioRisk project. Cardiovasc. J. Afr..

[101] Sriha Belguith A., Beltaief K., Msolli M.A., Bouida W., Abroug H., Ben Fredj M., Zemni I., Grissa M.H., Boubaker H., Hsairi M. (2018). Management of acute coronary syndrome in emergency departments: A cross sectional multicenter study (Tunisia) 11 Medical and Health Sciences 1117 Public Health and Health Services. BMC Emerg. Med..

[102] Abbasi S.H., Ponce de Leon A., Kassaian S.E., Karimi A.A., Sundin Ö., Soares J., Macassa G. (2012). Gender Differences in the Risk of Coronary Artery Disease in Iran. Iran. J. Public Health.

[103] Joshi P., Islam S., Pais P., Reddy S., Dorairaj P., Kazmi K., Pandey M.R., Haque S., Mendis S., Rangarajan S. (2007). Risk factors for early myocardial infarction in South Asians compared with individuals in other countries. J. Am. Med. Assoc..

[104] Allana S., Khowaja K., Ali T.S., Moser D.K., Khan A.H. (2015). Gender Differences in Factors Associated with Prehospital Delay Among Acute Coronary Syndrome Patients in Pakistan. J. Transcult. Nurs..

[105] Gender Differences in Presentation, Management and Outcomes among Patients with Acute Coronary Syndrome in Dhaka, Bangladesh. tctmd.com.

[106] Xavier D., Pais P., Devereaux P., Xie C., Prabhakaran D., Reddy K.S., Gupta R., Joshi P., Kerkar P., Thanikachalam S. (2008). Treatment and outcomes of acute coronary syndromes in India (CREATE): A prospective analysis of registry data. Lancet.

[107] Sigamani A., Kamath D., Xavier D., Pais P. (2013). New evidence for gender disparities in cardiac interventions: CREATE-ing some clarity [Internet]. Interv. Cardiol..

[108] Patel A., Vishwanathan S., Nair T., Bahuleyan C.G., Jayaprakash V.L., Baldridge A., Huffman M.D., Prabhakaran D., Mohanan P.P. (2015). Sex Differences in the Presentation, Diagnosis, and Management of Acute Coronary Syndromes: Findings from the Kerala-India ACS Registry. Glob. Heart.

[109] Cader F.A., Rahman A., Ullah M., Rahman M.A., Alam M.S., Nasrin S., Momen A., Kundu S.K., Chakraborty S., Bala P. (2018). Gender Differences in Clinical, Angiographic and Procedural Profiles between Young Patients with Acute Coronary Syndrome undergoing Percutaneous Coronary Intervention. Cardiovasc. J..

[110] Cader F.A., Rahman A., Rahman M.A., Zaman S., Arefin M., Reza A., Matin M.A., Afroz F., Hasnat A., Jafor M. (2018). Comparison of Short-term Outcomes of Percutaneous Coronary Intervention between Young Male and Female Patients with Acute Coronary Syndrome. Bangladesh Heart J..

[111] Galappatthy P., Bataduwaarachchi V., Ranasinghe P., Galappatthy G., Senerath U., Wijeyaratne C., Ekanayake R. (2020). Sex Difference in Risk Factors, GRACE Scores, and Management among Post-Acute Coronary Syndrome Patients in Sri Lanka. Cardiol. Res. Pract..

[112] Srichaiveth B., Ruengsakulrach P., Visudharom K., Sanguanwong S., Tangsubutr W., Insamian P. (2007). Impact of gender on treatment and clinical outcomes in acute ST elevation myocardial infarction patients in Thailand. J. Med. Assoc. Thail..

[113] Suttirut P., Panthong S., Kittipibul V., Lertsuwunseri V., Srimahachota S., Ariyachaipanich A. (2017). Impact of Gender on Outcomes Among Patients with Acute Coronary Syndrome Undergoing Primary Percutaneous Coronary Intervention in Southeast Asia. J. Am. Coll. Cardiol..

[114] Sella Y.O., Manistamara H., Apriliawan S., Lukitasari M., Rohman M.S. (2021). Characteristic differences of chest pain in male and female patients with acute coronary syndrome: A pilot study. J. Public Health Res..

[115] Bachelet B.C., Hyun K., D’Souza M., Chow C.K., Redfern J., Brieger D.B. (2022). Sex differences in the management and outcomes of non-ST-elevation acute coronary syndromes. Med. J. Aust..

[116] Worrall-Carter L., MacIsaac A., Scruth E., Rahman M.A. (2017). Gender difference in the use of coronary interventions for patients with acute coronary syndrome: Experience from a major metropolitan hospital in Melbourne, Australia. Aust. Crit. Care.

[117] Kam R., Cutter J., Chew S.K., Tan A., Emmanuel S., Mak K.H., Chan C.N.S., Koh T.H., Lim Y.L. (2002). Gender differences in outcome after an acute myocardial infarction in Singapore. Singap. Med. J..

[118] Levy M., Chen Y., Clarke R., Guo Y., Lv J., Yu C., Li L., Chen Z., Mihaylova B. (2022). Gender differences in use of invasive diagnostic and therapeutic procedures for acute ischaemic heart disease in Chinese adults. Heart.

[119] Hong J.S., Kang H.C. (2015). Sex Differences in the Treatment and Outcome of Korean Patients with Acute Myocardial Infarction Using the Korean National Health Insurance Claims Database. Medicine.

[120] Kawano H., Soejima H., Kojima S., Kitagawa A., Ogawa H. (2006). Sex differences of risk factors for acute myocardial infarction in Japanese patients. Circ. J..

[121] Kosuge M., Kimura K., Kojima S., Sakamoto T., Ishihara M., Asada Y., Tei C., Miyazaki S., Sonoda M., Tsuchihashi K. (2006). Sex Differences in Early Mortality of Patients Undergoing Primary Stenting for Acute Myocardial Infarction. Circ. J..

[122] Vaccarino V., Parsons L., Peterson E.D., Rogers W.J., Kiefe C.I., Canto J. (2009). Sex differences in mortality after acute myocardial infarction: Changes from 1994 to 2006. Arch. Intern. Med..

[123] Yan A.T., Yan R.T., Tan M., Fung A., Cohen E.A., Fitchett D.H., Langer A., Goodman S.G. (2007). Management patterns in relation to risk stratification among patients with non-ST elevation acute coronary syndromes. Arch. Intern. Med..

[124] Chest Pain MI Registry. https://cvquality.acc.org/NCDR-Home/registries/hospital-registries/chest-pain-mi-registry#:~:text=The%20Chest%20Pain%20%2D%20MI%20Registry%20offers%20a%20wealth%20of%20reporting,performance%20measures%20and%20quality%20metrics.

[125] Get with The Guidelines®–Coronary Artery Disease|American Heart Association. https://www.heart.org/en/professional/quality-improvement/get-with-the-guidelines/get-with-the-guidelines-coronary-artery-disease.

[126] Peterson E.D., Lansky A.J., Kramer J., Anstrom K., Lanzilotta M.J. (2001). Effect of gender on the outcomes of contemporary percutaneous coronary intervention. Am. J. Cardiol..

[127] Akhter N., Milford-Beland S., Roe M.T., Piana R.N., Kao J., Shroff A. (2009). Gender differences among patients with acute coronary syndromes undergoing percutaneous coronary intervention in the American College of Cardiology-National Cardiovascular Data Registry (ACC-NCDR). Am. Heart J..

[128] de Matos Soeiro A., de Barros e Silva P.G.M., de Castro Roque E.A., Bossa A.S., Biselli B., de Carvalho Andreucci Torres Leal T., de Almeida Soeiro M.C.F., Pitta F.G., Serrano C.V., Oliveira M.T. (2018). Prognostic Differences between Men and Women with Acute Coronary Syndrome. Data from a Brazilian Registry. Arq. Bras. Cardiol..

[129] Mariani J.A., Antonietti L., Tajer C.D., De Abreu M., Charask A., Silberstein M., Gagliardi J., Doval H.C. (2013). Diferencias de género en el tratamiento de síndromes coronarios agudos: Resultados del registro Epi-Cardio [Internet]. Rev. Argent. Cardiol..

[130] Pelletier R., Humphries K.H., Shimony A., Bacon S.L., Lavoie K.L., Rabi D., Karp I., Avgil Tsadok M., Pilote L. (2014). Sex-related differences in access to care among patients with premature acute coronary syndrome. CMAJ.

[131] Johnston N., Bornefalk-Hermansson A., Schenck-Gustafsson K., Held C., Goodman S.G., Yan A.T., Bierman A.S. (2013). Do clinical factors explain persistent sex disparities in the use of acute reperfusion therapy in STEMI in Sweden and Canada?. Eur. Heart J. Acute Cardiovasc. Care.

[132] Quesada O., Van Hon L., Yildiz M., Madan M., Sanina C., Davidson L., Htun W.W., Saw J., Garcia S., Dehghani P. (2022). Sex Differences in Clinical Characteristics, Management Strategies, and Outcomes of STEMI with COVID-19: NACMI Registry. J. Soc. Cardiovasc. Angiogr. Interv..

[133] Vogel B., Farhan S., Hahne S., Kozanli I., Kalla K., Freynhofer M.K., Jarai R., Kautzky-Willer A., Huber K. (2016). Sex-related differences in baseline characteristics, management and outcome in patients with acute coronary syndrome without ST-segment elevation. Eur. Heart J. Acute Cardiovasc. Care.

[134] Leifheit-Limson E.C., D’Onofrio G., Daneshvar M., Geda M., Bueno H., Spertus J.A., Krumholz H.M., Lichtman J.H. (2015). Sex Differences in Cardiac Risk Factors, Perceived Risk, and Health Care Provider Discussion of Risk and Risk Modification Among Young Patients with Acute Myocardial Infarction: The VIRGO Study. J. Am. Coll. Cardiol..

[135] Chandrasekhar J., Gill A., Mehran R. (2018). Acute myocardial infarction in young women: Current perspectives. Int. J. Women’s Health.

[136] Ya’qoub L., Lemor A., Dabbagh M., O’Neill W., Khandelwal A., Martinez S.C., Ibrahim N.E., Grines C., Voeltz M., Basir M.B. (2021). Racial, Ethnic, and Sex Disparities in Patients with STEMI and Cardiogenic Shock. JACC Cardiovasc. Interv..

[137] Ghadri J.R., Sarcon A., Jaguszewski M., Diekmann J., Bataiosu R.D., Hellermann J., Csordas A., Baumann L., Schöni A.A., Lüscher T.F. (2015). Gender disparities in acute coronary syndrome: A closing gap in the short-term outcome. J. Cardiovasc. Med..

[138] Neumann J.T., Goßling A., Sörensen N.A., Blankenberg S., Magnussen C., Westermann D. (2020). Sex-Specific Outcomes in Patients with Acute Coronary Syndrome. J. Clin. Med..

[139] Ruiz-Nodar J.M., Ferreiro J.L., Ribera A., Marsal J.R., García Acuña J.M., Agra Bermejo R., Raposeiras-Roubín S., Abu-Assi E., Cordero A., Bertomeu-González V. (2021). Sex differences in the management of patients with acute coronary syndrome: A population-based ecological cross-sectional study in Spain. REC CardioClin..

[140] Isorni M.A., Blanchard D., Teixeira N., Le Breton H., Renault N., Gilard M., Lefèvre T., Mulak G., Danchin N., Spaulding C. (2015). Impact of gender on use of revascularization in acute coronary syndromes: The national observational study of diagnostic and interventional cardiac catheterization (ONACI). Catheter. Cardiovasc. Interv..

[141] Johnston N., Jönelid B., Christersson C., Kero T., Renlund H., Schenck-Gustafsson K., Lagerqvist B. (2015). Effect of Gender on Patients With ST-Elevation and Non-ST-Elevation Myocardial Infarction Without Obstructive Coronary Artery Disease. Am. J. Cardiol..

[142] Pizzi C., Xhyheri B., Costa G.M., Faustino M., Flacco M.E., Gualano M.R., Fragassi G., Grigioni F., Manzoli L. (2016). Nonobstructive versus obstructive coronary artery disease in acute coronary syndrome: A meta-analysis. J. Am. Heart Assoc..

[143] Kunadian V., Qiu W., Lagerqvist B., Johnston N., Sinclair H., Tan Y., Ludman P., James S., Sarno G. (2017). Gender Differences in Outcomes and Predictors of All-Cause Mortality After Percutaneous Coronary Intervention (Data from United Kingdom and Sweden). Am. J. Cardiol..

[144] Spirito A., Gragnano F., Corpataux N., Vaisnora L., Galea R., Svab S., Gargiulo G., Siontis G.C.M., Praz F., Lanz J. (2021). Sex-based differences in bleeding risk after percutaneous coronary intervention and implications for the academic research consortium high bleeding risk criteria. J. Am. Heart Assoc..

[145] Bugiardini R., Badimon L., Manfrini O., Boytsov S., Božidarka K., Daullxhiu I., Dilic M., Dorobantu M., Erglis A., Gafarov V. (2014). Perspectives: Rationale and design of the ISACS-TC (International Survey of Acute Coronary Syndromes in Transitional Countries) project. Eur. Heart J. Suppl..

[146] Cenko E., Ricci B., Kedev S., Vasiljevic Z., Dorobantu M., Gustiene O., Knežević B., Miličić D., Dilic M., Manfrini O. (2016). Invasive versus conservative strategy in acute coronary syndromes: The paradox in women’s outcomes. Int. J. Cardiol..

[147] Vasiljevic- Pokrajcic Z., Mickovski N., Davidovic G., Asanin M., Stefanovic B., Krljanac G., Radosavljevic-Radovanovic M., Radovanovic N., Lasica R., Milanović S. (2016). Sex and age differences and outcomes in acute coronary syndromes. Int. J. Cardiol..

[148] Cenko E., van der Schaar M., Yoon J., Kedev S., Valvukis M., Vasiljevic Z., Ašanin M., Miličić D., Manfrini O., Badimon L. (2019). Sex-Specific Treatment Effects After Primary Percutaneous Intervention: A Study on Coronary Blood Flow and Delay to Hospital Presentation. J. Am. Heart Assoc..

[149] Cenko E., van der Schaar M., Yoon J., Manfrini O., Vasiljevic Z., Vavlukis M., Kedev S., Miličić D., Badimon L., Bugiardini R. (2019). Sex-Related Differences in Heart Failure After ST-Segment Elevation Myocardial Infarction. J. Am. Coll. Cardiol..

[150] Cepas-Guillen P.L., Echarte-Morales J., Flores-Umanzor E., Fernandez-Valledor A., Caldentey G., Viana-Tejedor A., Martinez Gomez E., Tundidor-Sanz E., Borrego-Rodriguez J., Vidal P. (2021). Sex-gender disparities in nonagenarians with acute coronary syndrome. Clin. Cardiol..

[151] De Carlo M., Morici N., Savonitto S., Grassia V., Sbarzaglia P., Tamburrini P., Cavallini C., Galvani M., Ortolani P., De Servi S. (2015). Sex-Related Outcomes in Elderly Patients Presenting with Non-ST-Segment Elevation Acute Coronary Syndrome: Insights from the Italian Elderly ACS Study. JACC. Cardiovasc. Interv..

[152] Piątek Ł., Wilczek K., Kurzawski J., Gierlotka M., Gąsior M., Poloński L., Sadowski M. (2020). Gender-related disparities in the treatment and outcomes in patients with non-ST-segment elevation myocardial infarction: Results from the Polish Registry of Acute Coronary Syndromes (PL-ACS) in the years 2012–2014. Arch. Med. Sci..

[153] Ruiz-Pizarro V., Ferrera C., Gómez-Polo J.C., Palacios-Rubio J., Rico-García Amado C., Fernández-Ortiz A., Viana-Tejedor A. (2019). Sex differences in treatment and prognosis of acute coronary syndrome with interventional management. Cardiovasc. Revasc. Med..

[154] Healy B. (1991). The Yentl syndrome. N. Engl. J. Med..

[155] Jin X., Chandramouli C., Allocco B., Gong E., Lam C.S.P., Yan L.L. (2020). Women’s participation in cardiovascular clinical trials from 2010 to 2017. Circulation.

[156] Cushman M., Shay C.M., Howard V.J., Jiménez M.C., Lewey J., McSweeney J.C., Newby L.K., Poudel R., Reynolds H.R., Rexrode K.M. (2021). Ten-Year Differences in Women’s Awareness Related to Coronary Heart Disease: Results of the 2019 American Heart Association National Survey: A Special Report from the American Heart Association. Circulation.

[157] Suero-Abreu G.A., Barajas-Ochoaa A., Perez-Peralta A., Rojas E., Berkowitz R. (2020). Assessment of the Effect of the Go Red for Women Campaign on Search Engine Queries for Cardiovascular Disease in Women. Cardiol. Res..

[158] Banerjee S., Aaysha Cader F., Gulati M., Capers Q. (2021). Racism and Cardiology: A Global Call to Action. CJC Open.

[159] Bairey Merz C.N., Andersen H., Sprague E., Burns A., Keida M., Walsh M.N., Greenberger P., Campbell S., Pollin I., McCullough C. (2017). Knowledge, Attitudes, and Beliefs Regarding Cardiovascular Disease in Women: The Women’s Heart Alliance. J. Am. Coll. Cardiol..

[160] Han J.E.D., Liu X., Bunce C., Douiri A., Vale L., Blandford A., Balaskas K. (2022). Tele-opHthalmology-enablEd and ARtificial Intelligence-ready referral pathway for coMmunity optomEtry referralS of retinal disease: The HERMES study—A Cluster Randomised Superiority Trial with a linked Observational Diagnostic Accuracy Study—NIHR Funding and Awards [Internet]. BMJ Open.

[161] Kannel W.B., Sorlie P., Mcnamara P.M. (1979). Prognosis after initial myocardial infarction: The Framingham study. Am. J. Cardiol..

